# Weed Classification Using Explainable Multi-Resolution Slot Attention

**DOI:** 10.3390/s21206705

**Published:** 2021-10-09

**Authors:** Sadaf Farkhani, Søren Kelstrup Skovsen, Mads Dyrmann, Rasmus Nyholm Jørgensen, Henrik Karstoft

**Affiliations:** Department of Electrical and Computer Engineering, Aarhus University, 8200 Aarhus, Denmark; ssk@ece.au.dk (S.K.S.); madsdyrmann@ece.au.dk (M.D.); rnj@agrointelli.com (R.N.J.); hka@ece.au.dk (H.K.)

**Keywords:** transformer, slot attention, explainable neural network, fusion rule, weed classification, weed identification key, precision agriculture

## Abstract

In agriculture, explainable deep neural networks (DNNs) can be used to pinpoint the discriminative part of weeds for an imagery classification task, albeit at a low resolution, to control the weed population. This paper proposes the use of a multi-layer attention procedure based on a transformer combined with a fusion rule to present an interpretation of the DNN decision through a high-resolution attention map. The fusion rule is a weighted average method that is used to combine attention maps from different layers based on saliency. Attention maps with an explanation for why a weed is or is not classified as a certain class help agronomists to shape the high-resolution weed identification keys (WIK) that the model perceives. The model is trained and evaluated on two agricultural datasets that contain plants grown under different conditions: the Plant Seedlings Dataset (PSD) and the Open Plant Phenotyping Dataset (OPPD). The model represents attention maps with highlighted requirements and information about misclassification to enable cross-dataset evaluations. State-of-the-art comparisons represent classification developments after applying attention maps. Average accuracies of 95.42% and 96% are gained for the negative and positive explanations of the PSD test sets, respectively. In OPPD evaluations, accuracies of 97.78% and 97.83% are obtained for negative and positive explanations, respectively. The visual comparison between attention maps also shows high-resolution information.

## 1. Introduction

Weeds compete with crops to capture sunlight and take up nutrients and water; this competition leads to significant yield losses around the world every year [1]. Furthermore, there are considerable indirect negative externalities that should be taken into consideration when combating weeds [2]. Currently, the use of conventional weed control methods usually results in soil erosion, global warming, and human health problems [3,4,5,6]. Weeds are usually not distributed evenly across farmlands. Therefore, weed management could be greatly improved by collecting information about the location, type, and amount of weeds in an area [7].

In general, there are three primary weed management strategies: biological, chemical, and physical [8]. Biological weed management refers to weed control through the use of other organisms, such as insects or bacteria, to maintain weed populations at a lower level [9]. Biological weed control is, however, a prolonged procedure that reduces the growth of a specific species. Selective chemical weed management using an autonomous and unmanned vehicle is one solution for controlling the weed population and requires the use of considerably lower contamination doses [10]. In the physical approach, weeds are controlled without herbicide; this is typically accomplished through the use of mechanical tools. Physical weed control requires extra precision in the detection of weeds, as non-selective and incorrect weed detection can harm the crop.

In physical and chemical methods, weed management is conducted in two steps: capturing images in the field and weed detection/classification [11]. The earlier step can feasibly be carried out through the use of new imaging technologies. In the second step, however, collecting and labeling data is a time-consuming and error-prone procedure, especially in agricultural areas where many different kinds of plants are mixed in [12,13,14]. In artificial neural network (ANN) modeling, it is possible to determine imprecise temporal and spatial parameters [15,16]. Thus, autonomous weed management methods combined with computer vision approaches could help farmers to detect and classify weeds and consequently improve weed management and decision-making [17,18]. Thus, the application of an accurate weed classification method plays a critical role in precise farming, helping to determine the weed-combating approach used, maximize crop yields, and improve economical returns [19,20,21,22].

CNNs have shown promising performance for image classification, including agricultural applications. However, one of the main challenges with deep neural networks (DNNs) is the lack of explanation, known as the *black-box* problem, concerning the human perception of the model’s logic within the classification [23]. Therefore, an interpretable map is an efficient means of explaining the model’s prediction as well as understanding the data better.

To mitigate the aforementioned challenges, explainable artificial intelligence (XAI) is proposed to present a better explanation of *black-box* DNN models [24]. In classification methods based on XAI, the model identifies the class prediction and highlights the critical data content to draw attention to a given decision. Therefore, the models are also called attention models.

In agriculture, the model’s explanation map supports a research area called the weed identification key (WIK), which is mainly adopted to discriminate species with a higher accuracy [25]. WIKs assist agronomists in classifying both common and uncommon features between species with an acceptable level of accuracy. Therefore, the model’s transparency helps us to create and understand the WIKs perceived by the model.

Positive and negative explanation maps, which explain why a model does or does not classify an image into a corresponding category, introduce both mutual and distinctive perceptible features from different classes. The negative explanation is especially informative in classification problems with high similarities between classes, such as in agricultural datasets [26].

Conventional WIKs include both positive and negative explanations simultaneously. In computer vision problems, self-attention transformers are utilized to discriminate the locations of objects. According to [27], the slot attention module includes multi-head attention blocks with dynamic weights [28,29]. Slot attention describes the latent features of DNNs by training a set of abstract representations, called slots, for different classes. In a slot attention module, discriminative object regions will be extracted without the need to use humans for supervision. The slot attention, however, will have a low resolution due to the poor resolution of the DNN’s latent features [26].

In this paper, two agricultural datasets are employed in the analysis: the Plant Seedlings Dataset (PSD) [30] and the Open Plant Phenotyping Database (OPPD) [31]. Both datasets have a weed species-annotated bounding-box for each plant. To improve the resolution of the slot attention with high-level semantics and fine details, a multi-resolution mechanism is adopted here that is based on the slot attention module. Afterwards, to manipulate different feature layers’ impacts on the resulting attention map, a weighted mean approach is used to combine multi-resolution maps regarding their saliency. Three main aspects are used for creating the slot attention in agricultural applications, and the proposed model is evaluated based on them: (1) the resolution of the attention map, (2) the size of the area covering the object, and (3) the features of the weed species that cause the model to not classify the weed as another class (hereafter called negative explanation).

The proposed framework for multi-resolution slot attention and the proposed weighted average method in this paper are described in Section 2. Then, in Section 3, the results are elaborated within two different setups. Lastly, the discussion and conclusion are provided in Section 4 and Section 5, respectively.

## 2. Materials and Methods

### 2.1. Utilized Datasets

The model was trained and tested with two different datasets to evaluate how well it could support attention on mutual features. Hence, two plant seedling datasets, PSD and OPPD, were employed in this paper. Differences in the growing medium were used to evaluate the proposed model on agricultural datasets with changing settings.

In Table 1, the European and Mediterranean Plant Protection Organization (EPPO) labels for the species utilized in this paper are shown. Monocot and dicot species are represented by M and D, respectively, in Table 1.

The PSD contains images of 960 unique plants across 12 plant species in several growth stages with a ground sampling distance of 10 pixels per mm [30]. The camera (Canon 600D) was placed at a 110–115 cm distance above the soil surface. Plants in the PSD are grown indoors with even illumination conditions. The surface of the soil in the PSD is covered with stones to avoid green indoor moss artifacts and to ease the distinction between plants and the background. There is no specific plant color variation in the PSD. In the PSD, weed species are detected and cropped out.

The original OPPD is comprised of 64,292 unique crop plants. These plants include 47 different species in multiple growth stages with a ground sampling distance of 6.6 pixels per mm [31]. In our work, we only considered growth stages and species that are common in the PSD. Therefore, 21,653 and 5393 plant images are utilized as training and test sets here, respectively. Images were illuminated using a ring flash to ensure consistent light conditions during the image acquisition. The OPPD was able to better capture the naturally occurring variability in the plant morphology of the species in abnormal conditions. To meet this goal, plants were grown with different amounts of water and levels of nutrition stress. As with the PSD, there is only one plant per image for training and testing the model.

**Figure 1:**
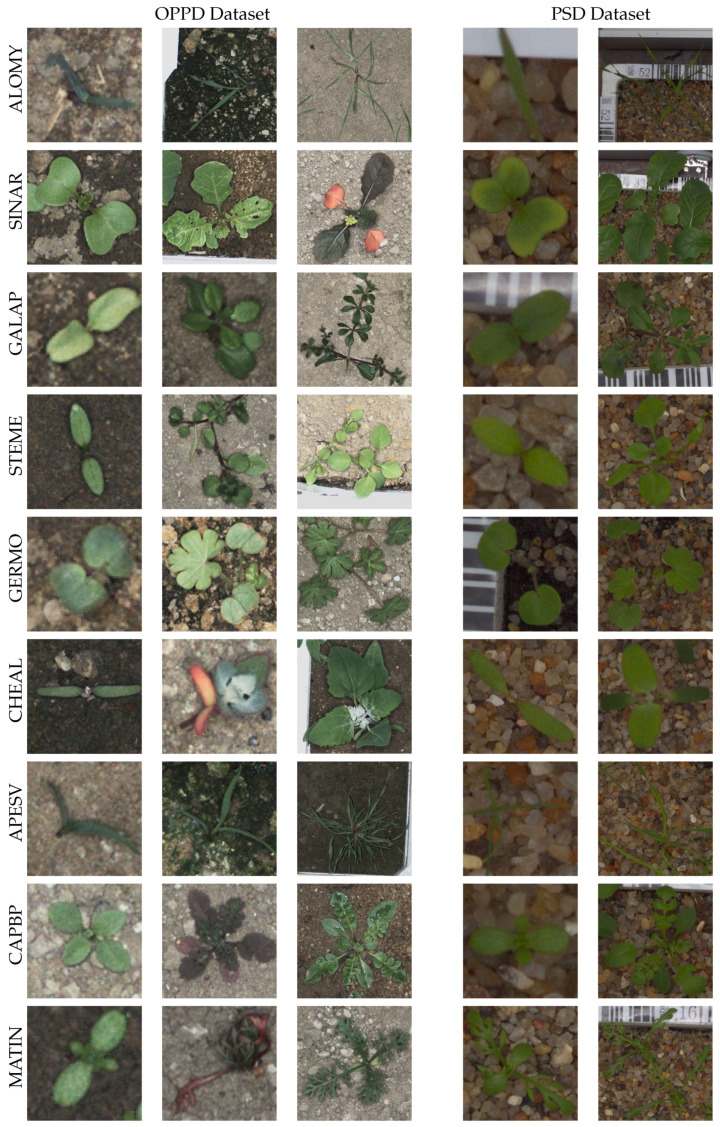
Examples of nine common species from the OPPD and PSD samples during different maturity stages, from left to right. OPPD samples were also selected from non-stressed and stressed samples.

Figure 1 shows different samples from species that are common to both the PSD and OPPD, respectively. Images are sorted from the left to right according to the growth stage. Three samples are shown for the OPPD and two for the PSD, since growth stage diversity is higher in the OPPD. There are multiple images for each plant in the growing procedure. The images depicted in Figure 1 were resized to a common resolution. Moreover, the samples in the training and test sets were randomly divided into proportions of 80% and 20%, respectively. The training and test samples were randomly separated for each image.

There are nine mutual species in the PSD and OPPD. Twelve species from the PSD were utilized in experiments when the PSD was employed for both training and testing. Otherwise, only the nine common species of the PSD and OPPD were fed into the network. The two datasets have different illumination conditions. For instance, there is a bright area around the terminal bud in the later growth stages of *CHEAL*, which is a deterministic feature. However, this feature is more apparent in the OPPD than in the PSD due to the illumination. Therefore, the combination of these two datasets could assist us in finding which features were brought out by the model and whether the absent features were essential.

It is necessary to mention that the scale of the images is varied due to the data augmentation technique (explained in Section 2.3) applied to the training set. Therefore, the differences in resolution between the two datasets cause no serious problem. On the other hand, the model’s generalizability was examined under changing light, acquisition, and growth conditions. We recommend that the reader review [30,31] if more details about the data acquisition process used in the PSD and OPPD are required.

### 2.2. Neural Network Architecture

**Figure 2:**
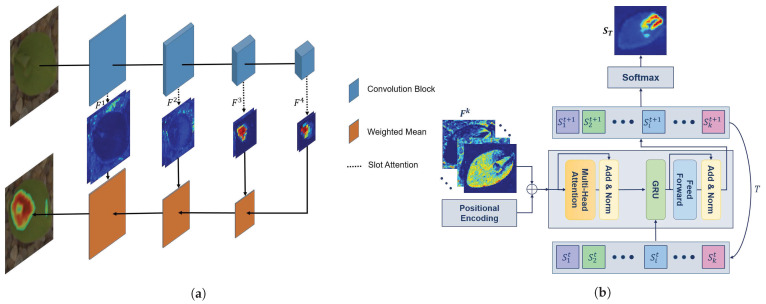
The proposed architecture for plant classification using the slot attention module. (**a**) The overall architecture for extracting features using convolutional blocks (in blue), including obtaining the highlighted attention areas from different convolutional blocks and combining multi-resolution slot attention to generate the final attention map (orange blocks). (**b**) The slot attention module applied to K-class weed classification using the transformer concept. Slots are depicted as $S_{i}^{t}$ for class *i* in iteration *t*.

The overall framework of the proposed pyramid representation—hereafter called high-resolution attention—was inspired by feature pyramid networks [32,33]. By extracting features from different levels, a high-resolution representation of the attention map was achieved (Figure 2).

The RGB input image is passed through a DNN to extract features at multiple depths and spatial resolutions (Figure 2a). The extracted features are then passed through the slot attention module (Figure 2b). The slot attention module mainly consists of a transformer. Ultimately, the extracted attention maps gained for other classes from different resolution levels are merged to obtain the high-resolution attention map as the output.

#### 2.2.1. Slot Attention

The slot attention is generated based on the feature regions with a great explainability of the class. The impact of different regions is formulated using positional encoding in Figure 2b. In this section, we illustrate not only the attention mechanism utilized [27], but also the method proposed to be used for extracting multi-resolution attention maps.

In Figure 2, the features were first extracted from different levels $F^{n}$ of the backbone. *n* depends on the number of spatial downsampling processes used in the DNN, which was four in the ResNet50 [34] adopted in this study. Then, $F^{n}$s were individually passed through the slot attention module to extract highlighted regions. Slot attention based on a transformer is an iterative module with *K* slots, where each slot describes a class in a *K*-classification problem. Through extracted features and positional encoding, the slots are trained to present maps with a high ability to explain the object. Slots are shown by $S_{i}^{t}$ and randomly initialized using a Gaussian distribution.

In the multi-head attention block shown in Figure 2, there are three main learnable vectors: keys (*k*), queries (*q*), and values (*v*) [35]. The *q* are the slots $S_{k}$ updated within *T* iterations. According to [27], the slots are trained to be sufficiently precise after three iterations. While *q* is formed based on the labels used, *k* and *v* are based on the inputs. The higher the similarity gained between *q* and *k* is, the better the model has been trained with respect to precision of explanation:

(1) $$U^{t+1}=<Softmax{\left(\frac{1}{\sqrt{D}}<k\left(inputs\right),q{\left(slots\right)}^{T}>\right)}^{T},v\left(inputs\right)>,$$

where Equation (1) is the multi-head attention block shown in Figure 2; *D* is the common dimension space between three vectors *q*, *k*, and *v* utilized as a normalization term; and $U^{t+1}$ is the updated slots obtained in iteration *t*. The inner product <.,.> of the vectors is computed to find the vectors’ similarities. Softmax is then applied to normalize the attention maps and suppress the attention gained for the other classes. Then, a gated recurrent unit (GRU) is utilized to update the slots [36]. GRU is a learnable recurrent function that is used for updating slots with the aggregated updates and previous slots. In [27], the investigations show improvements in the model’s performance when a multi-layer perceptron (MLP) is adopted after the GRU,

(2) $$S^{t+1}=MLP\left(GRU\left(S^{t},e\cdot U^{t+1}\right)\right),S^{t}=\left[S_{1}^{t},S_{2}^{t},\ldots ,S_{k}^{t}\right],$$

where $S^{t}$ and $S^{t+1}$ are the previous and updated slots, respectively. Therefore, all the slots are updated in each iteration. To easily switch between positive and negative explanations, the sign parameter *e* is determined. A comprehensive description of the negative explanation and Equation (2) is provided in the study of [26].

Instead of interpolating the last layer features to gain an attention map with the same input dimension, we applied the slot attention after four convolutional blocks in ResNet50. Afterwards, slots from different layers with different resolutions were combined using a fusion rule described in the next section.

#### 2.2.2. Fusion Rule

In slot attention, deeper layers have a sparse but high accuracy regarding the object explanation, albeit with a lower spatial resolution. On the other hand, shallower layers have a high spatial resolution with a lower accuracy regarding object localization. When combining different layers of slot attention, the degree of certainty should impact the dedicated weight of the fused attention maps. The higher the average values of the slots are, the higher the model’s certainty will be with regard to localization. Therefore, a slot attention map with higher average values should have a higher impact on the fused attention map. The following equation was used for this purpose to combine different layer attention maps:

(3) $$S_{F}=\sum_{l=1}^{n}\frac{W_{l}}{\sum_{j=1}^{n}W_{j}}\cdot S^{l},$$

where $W_{l}$ is the summation of elements in the updated slots for the *l*th layer of the backbone. In other words, the attention map with the highest $W_{l}$ had the greatest effect on the fused slots $S_{F}$. In Figure 2, the fusion rule is represented by an orange block. Therefore, the final attention map was formed based on the combination of shallow layers with a high precision and deep layers with a high resolution. The proposed weighted mean approach preserves the highlighted areas through the use of upsampling.

#### 2.2.3. Loss

Two loss functions were required for this problem: one for the classification and the other for the attention. For the classification, the cross-entropy ($L_{CE}$) of the deepest layer of the backbone was computed, ref. [26] presents SCOUTER loss, defining how large the attention area should be through the formula:

(4) $$L_{SCOUTER}=L_{CE}+\lambda W,$$

where *W* is the sum of elements in the slots gained from different backbone layers controlled by the hyperparameter λ. λ is adjusted based on how broad the attention areas are in the specific dataset.

### 2.3. Parameter Setting

Input images in both PSD and OPPD have square dimensions. Input images were first resized to 360×360 pixels with bilinear interpolation to balance images with different dimensions at different growth stages. Then, ResNet50 [34] was used as the backbone in order to extract the latent features. There are four convolutional blocks in ResNet50. Thus, four slot attention modules were implemented on intermediate features to merge the attention maps created based on their saliency. The model is implemented by PyTorch v1.7. The model was pretrained using ImageNet [37]. The batch size was 32, the initial learning rate was $10^{-4}$, and AdamW [38] was utilized as the optimizer. The attention was shown in positive and negative explanations. In Equation (4), λ was set to 2 in all evaluations based on trial and error. The number of iterations used for the slot attention was set to three. Additionally, the model was trained in 80 epochs. In the training procedure, the model was trained using multiple training processes on four GPUs (48 GB).

Translation, rotation, scaling, shear, cut-out, image corruption, Gaussian noise, and color space-changing methods were utilized as data augmentation techniques (color augmentation was only employed for generating results in Section 3.3). The translation (along the x and y axes), rotation, scaling, and shear were randomly selected within [−0.1, 0.1] of the input’s dimension, [$-10^{\circ }$, $10^{\circ }$], [0.8, 1], and [$-20^{\circ }$, $20^{\circ }$], respectively. Only a few data augmentation methods were randomly applied to the data each time in order to avoid significant variations in the images.

## 3. Results

This section is ordered into an exploration of attention maps from different backbone layers, an evaluation of the PSD, and a cross-evaluation of the OPPD and PSD.

### 3.1. Multi-Resolution Attention

**Figure 3:**
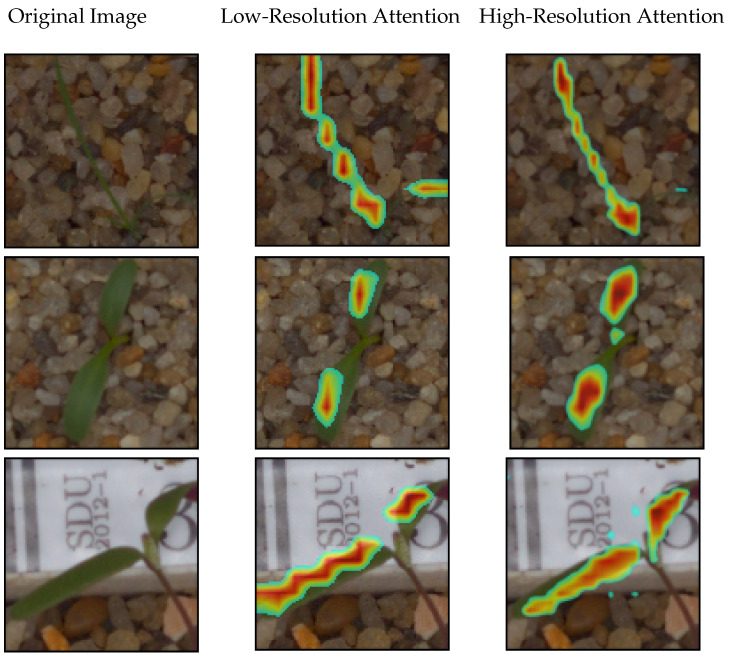
Comparison between the low- and high-resolution predicted attention map for three samples from different classes.

Figure 3 shows the attention maps gained using three examples from different classes (narrow and broad leaves). The attention map was utilized as the alpha channel, with areas with values close to zero neglected by applying a threshold. The original images are shown to give a better view of where weeds are located. Low-resolution attention was obtained by using only the backbone’s last layer of slot attention. High-resolution attention was obtained by applying the weighted average to the attention maps gained from different levels.

The attention map gained from only the last layer of the backbone is highly precise in terms of discriminating the salient features of weeds, as shown in the middle column of Figure 3. In the low-resolution attention map, highlighted areas were roughly distributed along the horizontal and vertical axes due to the interpolation (the middle column in Figure 3). Moreover, attention spots in the low-resolution map were not placed precisely on the weed. Contrarily, the high-resolution attention map was distributed smoothly along the plant (the right column in Figure 3).

It is worth mentioning that the predicted attention was partly placed on the background in some cases of high-resolution attention (such as the last row in Figure 3). This phenomenon was likely due to the impact of shallower layers on the combined attention map. This result could also be related to noisy backgrounds, blurred features, etc. For example, in the last row of Figure 3, the high-resolution attention map also points to stones and the box in the background.

**Figure 4:**
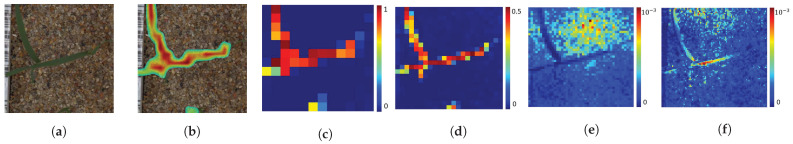
The impact of multi-resolution attention maps. (**a**) The original image and (**b**) the weighted averaged attention map. (**c**) 4th layer, (**d**) 3rd layer, (**e**) 2nd layer, and (**f**) 1st layer slot attention gained from the backbone layers. The bluish areas in (**b**) were filtered to improve the clarity of the visualization.

Additionally, attention maps from different layers on a weed-specific sample are shown in Figure 4. All slots from different layers are scaled up in Figure 4. The two last layers (Figure 4e,f) had an excellent resolution compared with the slot attention from the other layers (Figure 4c,d). Normalized heatmaps from various slots are presented here in order to give a better demonstration of each layer’s attention. The scale bar for each slot is presented alongside it. It is necessary to mention that the legends are not directly comparable between figures. The 4th and 3th layers’ weights, referred to as $W_{l}$ in Equation (3), were considerably more important than the 2nd and 1st layers. In other words, while the attention maps extracted from the deeper layers (Figure 4c,d) had a higher accuracy in identifying plants, the attention maps from shallower layers (Figure 4e,f) had lower attention weights for the whole image.

The weighted average fusion rule provides a balance between accurate, low-resolution attention from the last layer and inaccurate, high-resolution attention from the first layer. In Figure 4b, the attention map has a multi-directional explanation from shallower layers with a high accuracy in detecting weeds from deeper layers simultaneously. Therefore, the distribution of the attention maps was enhanced and developed to provide precise, omnidirectional attention maps. The omnidirectional attention map was creating using high-resolution attention maps from shallower layers.

### 3.2. Evaluations on the PSD

**Figure 5:**
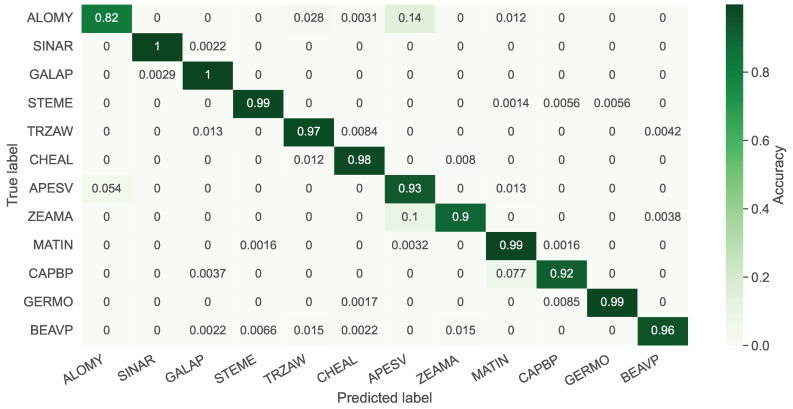
The average confusion matrix for the negative explanation of the PSD test set with 12 classes. The overall accuracy gained was 95.42%.

In this section, all 12 species in the PSD are employed for training and inference. In Figure 5, the average confusion matrix for the test set is shown for the negative explanation across ten repeats. The negative attention helps us to explicitly understand the data better. The average is then computed, since the model performance slightly changes for the random data augmentation and weight initialization. All samples visualized in attention matrices were selected from correctly classified instances.

In Figure 5, the average accuracy is 95.42%. The diagonal of the matrix has a more than 90% accuracy for all samples, except *ALOMY*. *ALOMY* and *APESV* are both monocots (narrow leaves), and it is hard to discriminate them using an agronomist. Since *APESV* comprises more samples than *ALOMY*, the model presented a clear bias towards misclassifying monocot samples as *APESV* when the uncertainty is high. Additionally, the model showed a clear tendency to classify *ZEAMA* (also monocot) as *APESV*. However, *ZEAMA* has a particular feature in earlier growth stages, making it easier for the model to identify it than *ALOMY*. Therefore, the model has a higher certainty for *ZEAMA*, particularly in the earlier growth stages.

**Figure 6:**
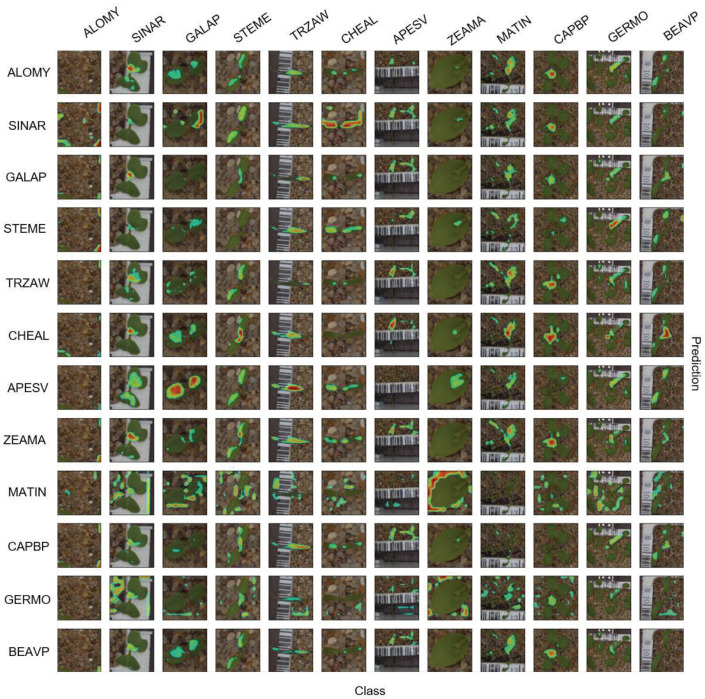
Negative attention matrix for PSD dataset with 12 classes. Columns are classes and rows are model predictions. The attention matrix’s diagonal has remarkably less attention, since the model classifies using the negative loss value in Equation (2).

In Figure 6, the attention confusion for the negative explanation is shown. It is expected that the highlighted areas will be absent in the diagonal, while the non-diagonal images will have meaningful distinctive attention areas.

The feature is well represented by the highlighted area, which is used for predicting *APESV* for *ZEAMA*. In Figure 6, the highlighted spots on the background were supposed to be generated for two reasons: (i) the scale of the stones varied regarding the growth stage (input images were re-scaled to 360×360) and the background had remarkable impacts in classes with small changes across different growth stages, and (ii) the positive layer’s weights were on the background while the negative layer’s weights were on the foreground.

**Figure 7:**
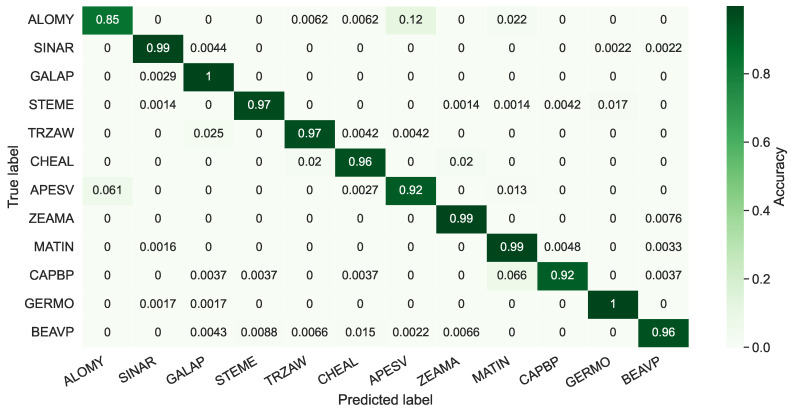
Confusion matrix for the positive explanation of the PSD test set with 12 classes. The average gained accuracy was 96%. The diagonal with a dark heatmap is desirable.

The positive confusion matrix is shown in Figure 7, which led to a similar trend as that for the negative explanation. The non-diagonal predictions for the same class are helpful for understanding which features were missed in the dataset or which species had higher similarities that made the model uncertain. Therefore, a high number of doubtful species were recognized and could be utilized as an alarm in the other field classification.

**Figure 8:**
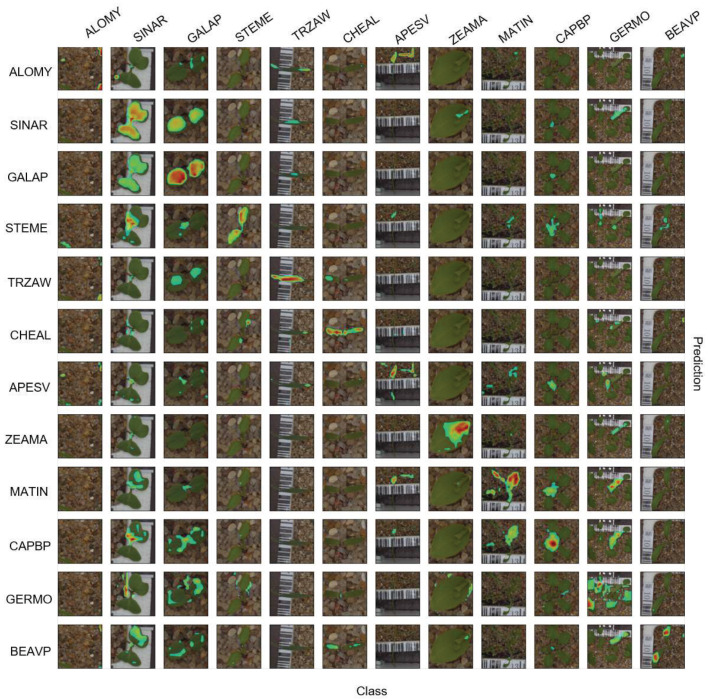
The positive attention matrix for the PSD test set with 12 classes. The diagonal images bold out the particular features that the model uses for the classification.

The same samples in the negative explanation are selected for the positive explanation in Figure 8. The diagonal attention areas show which part of the plant has a significant weight in classification during training. In other words, the positive explanation emphasizes species patterns that are necessary for the model. In class *ZEAMA*, for instance, the highlighted area shows the particular part that is unique in the class and not the whole leaf. Comparing Figure 7 with Figure 5, it can be seen that the accuracy of the class *ZEAMA* improved by approximately 9% from the negative to the positive explanation. The reason for this was that *ZEAMA* has similarities to both monocots and dicots (broadleaves). As a result, it was simpler for the network to reveal the unique feature for *ZEAMA* (in Figure 8) in the negative explanation (in Figure 6). This also reveals the accuracy improvement from the negative to positive explanation.

In Figure 8, the model came with different parts of plants in different classes or growth stages, depending on the similarities between species. For instance, while the model’s attention was on the whole leaves for *GALAP*, as an example of a case that is difficult to classify during early growth stages, the main attention was on the center of the plant for *CAPBP*, as an example of a case that is easier to classify in the later growth stages.

### 3.3. Evaluations on PSD and OPPD

**Figure 9:**
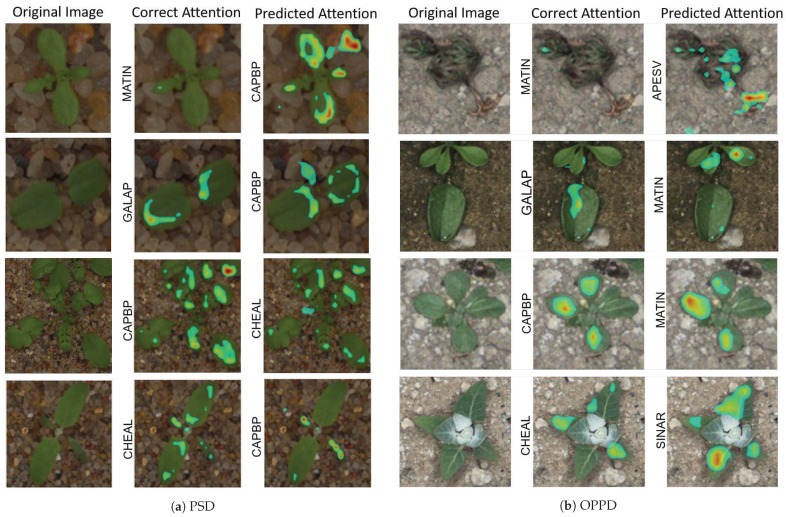
Misclassified samples in cross-dataset evaluation. In (**a**), the model was trained on the OPPD with positive attention, while the inference was trained on the PSD. Conversely, in (**b**), the model was trained on the PSD with positive attention, while the inference was trained on the OPPD.

In this section, the model was trained on the PSD and inferenced on the OPPD as a cross-dataset evaluation. In Figure 9, eight misclassified samples are shown through cross-dataset evaluation. For each sample, correct and predicted positive attention maps are depicted on the original image. The label for each slot attention is presented on the left side of the image. Four class species are shown for two cross-dataset evaluations: (i) in Figure 9a, the model was trained on the OPPD and evaluated on the PSD, while (ii) in Figure 9b the model was trained on the PSD and tested on the OPPD.

Classes *CAPBP* and *MATIN* look similar in their earlier growth stages, which made prediction harder. Furthermore, samples of stressed species from the OPPD were misclassified in most cases. For instance, a stressed sample from class *MATIN* is shown in Figure 9b from the OPPD which was predicted as class *APESV*.

In Table 2, a comparison between the use of the proposed method and state-of-the-art methods on the PSD and OPPD is shown. The proposed method in this study was evaluated with both a positive explanation, *Ours+*, and a negative explanation, *Ours−*. For the PSD, two other state-of-the-art methods are compared in Table 2.

In Table 2, the proposed method was found to outperform the previous methods in both OPPD and PSD evaluations, ref. [39] conducted the training with a five-fold cross-validation of the PSD using EfficientNet. The number of parameters used was lower in our methods (the negative and positive attention models) in spite of the use of the multiple slot attention module, since the fully connected layer is omitted. However, the number of parameters utilized in [41] is considerably lower than that in the attention method proposed in this paper.

The OPPD was published quite recently and only one applied method is given as a comparison in Table 2. In the OPPD study conducted by [41], the SE-module is implemented for classification. The SE-module is a multi-scale fusion approach that does not utilize attention. The proposed method outperformed the method described in the study by [41] in terms of accuracy.

Instances from different growth stages of the class *CHEAL* are presented in Table 3 to emphasize the importance of contrast and color space in classification. The result in Table 3 was gained by a model that had been both trained and tested on the OPPD. In the first growth stage, attention was also paid to leaves (the last row). However, the attention was attracted to the center in later growth stages (the first and middle rows).

The impact of growth stage on *CHEAL* is shown in Table 3. In the OPPD, the class *CHEAL* was a prominent feature in the later growth stages; there are white hairs on the leaves that are more obvious in the center of plants. In the PSD, however, the whitish domain is less visible due to the different brightness and contrast. Therefore, the attention gained from the training and inference with the PSD and the OPPD, respectively, highlighted areas over leaves, not over stems.

The model shows the leaves for monocot species in Table 3—i.e., *ALOMY* and *MATIN*—since the broad leaves are distinctive areas in the later growth stages. For dicot species, the white center area gained the model’s attention.

## 4. Discussion

The proposed model presented a high-resolution attention map of weed species; the map enabled us to better perceive the model’s decision [42]. In the previous transformer-based methods, the resolution of the attention map was low due to the interpolation applied for resizing the attention map from 12×12 to 360×360 [43]. This challenge was mitigated in the approach proposed in this paper by providing a multi-layer attention mechanism. In general, the model has a lower certainty regarding attention in the first few layers [44,45], but its precision is higher. Therefore, the proposed algorithm merged multi-layer attention maps from different layers to generate a precise high-resolution representation that included principle features for weed discrimination.

The positive explanation maps help us to differentiate weed species during the early growth stages and are frequently utilized in transformers [42,46]. Moreover, the negative explanation maps support the model’s classification, particularly during the mature growth stages, where the dissimilarities between species are substantial [26]. Moreover, the model’s uncertainty should help farmers to decide which species should be reconsidered during weed management [47].

In terms of statistical comparison, the proposed model outperformed the state-of-the-art methods using positive attention, as illustrated in Table 2. The performances of the proposed model showed slight improvements compared with those shown in the study by [40]. This is likely due to the attention explanation, better data augmentation, tuning of the hyper-parameters, etc. The attention loss also showed improvements in terms of classification for the positive and negative explanations of the PSD.

The model’s challenges in cross-dataset evaluation (Section 3.3) showed that a model applied to one agricultural dataset might not be robust on the other datasets [48]. The proposed model presented interpretable information about the differences between the two datasets, which made the model unable to classify properly. Moreover, only diversity was not sufficient to improve the performance, since the model that was trained with the OPPD and had a wider variety still struggled when applied to the PSD. Nevertheless, the proposed method should help us determine what areas showed significant differences between the two datasets. Therefore, there should be a better explanation as to why the model achieved a lower accuracy during the classification. The cross-dataset evaluation also highlighted the necessity of understanding the data better during the training and test phases in DNNs.

In the cross-dataset evaluation, three characteristics that will be considered in future research were not taken into account:

1. Growth stage;
2. Partial or heavy occlusion;
3. Partial plant appearance.

The model’s performance is expected to be improved when a growth stage label is also given to the model due to species variation in different growth stages [13,49]. Furthermore, two critical factors observed from Figure 9 were the impact of occlusion and partial appearance due to the classification. For instance, class *GALAP* showed a partial appearance in Figure 9b (where only half of the plant is visualized), while class *CAPBP* showed partial occlusion due to the neighboring plant in Figure 9a. A great quantity of real in-field annotated images would support our knowledge about the model’s performance regarding the existence of occlusion, stress, neighboring plants, etc. In conclusion, the characteristics mentioned above should be investigated in future research.

## 5. Conclusions

In this paper, a high-resolution attention architecture was proposed in order to improve the resolution and location of highlighted weed areas in weed management. The resulting explanation is a foolproof approach for interpreting the similarities and dissimilarities between different weed species through automated weed control. By understanding the black-box model better, we were able to gain more transparency regarding the model’s classification of different weed species through maturation. Therefore, self-attention maps from different layers of a ResNet model were extracted to improve the attention precision. The proposed method was able to simultaneously preserve the accuracy from deeper layers and develop the resolution using shallower layers. In addition, this explanation is useful when studying the generalizability of a model for cross-dataset evaluations. The proposed precise and high-resolution attention map was able to explain the datasets better in terms of their visual aspect. Furthermore, the high-resolution attention map highlighted different patterns in a species through various growth stages. The influence of growth stage on attention maps through weed classification is a matter that should be investigated in future studies.

## Figures and Tables

**Table 1:** EPPO code and English name of the species utilized in this paper.

EPPO Code	English Name	Mono/Dicot
ALOMY	Black grass	M
APESV	Loose silky-bent	M
BEAVP	Sugar beet	D
CAPBP	Shepherd’s purse	D
CHEAL	Fat hen	D
GALAP	Cleavers	D
GERMO	Small-flowered crane’s bill	D
MATIN	Scentless mayweed	D
SINAR	Charlock	D
STEME	Common chickweed	D
TRZAW	Common wheat	D
ZEAMA	Maize	M

**Table 2:** The comparison between the use of multi-resolution attention on the PSD and OPPD test sets with the state-of-the-art methods.

	Dataset	Accuracy (%)	Parameters (M)
**EffNet** [39]	OPPD	95.44	7.8
**ResNet50** [40]	OPPD	95.23	25
**Ours−**	OPPD	95.42	23.98
**Ours+**	OPPD	**96.00**	23.98
**SE-Module** [41]	PSD	96.32	1.79
**Ours−**	PSD	97.78	23.54
**Ours+**	PSD	**97.83**	23.54

**Table 3:** Positive and negative explanation of the class *CHEAL* by the model trained and tested on the OPPD dataset. Images are sorted in order of increasing growth stage. The model had different highlighted areas and understandings of *CHEAL* in different growth stages.

Original Image	Positive Attention	Negative Attention							
		ALOMY	SINAR	GALAP	STEME	GERMO	APESV	CAPBP	MATIN
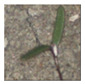	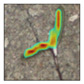	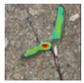	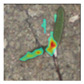	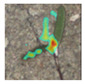	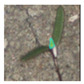	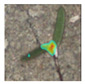	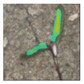	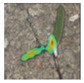	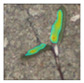
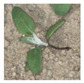	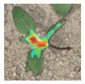	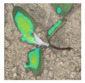	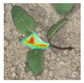	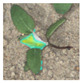	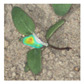	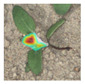	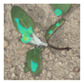	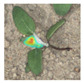	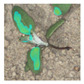
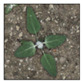	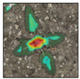	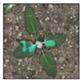	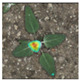	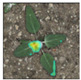	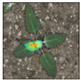	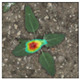	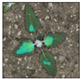	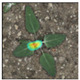	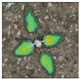

## Data Availability

Publicly available datasets were analyzed in this study. The data [30,31] can be found from: https://vision.eng.au.dk/plant-seedlings-dataset/ and https://gitlab.au.dk/AUENG-Vision/OPPD, accessed on 17 September 2021, respectively.

## Abbreviations

The following abbreviations are used in this paper:

CNN	Convolutional neural network
D	Dicot
DNN	Deep neural network
EPPO	European and Mediterranean Plant Protection Organization
GRU	Gated recurrent unit
M	Monocot
MLP	Multi-layer perceptron
OPPD	Open Plant Phenotyping Dataset
PSD	Plant Seedlings Dataset
ReLU	Rectified linear unit
WIK	Weed identification key
XAI	Explainable artificial intelligence
